# Supplementary material for: TFF3 interacts with LINGO2 to regulate EGFR activation for protection against colitis and gastrointestinal helminths
Source: Nat Commun. 2019 Sep 27;10:4408. doi: 10.1038/s41467-019-12315-1 (PMC6764942; doi:10.1038/s41467-019-12315-1)
Supplement: Supplementary file 1 — Supplementary Information [file 41467_2019_12315_MOESM1_ESM.pdf]

Supplementary information

TFF3 interacts with LINGO2 to regulate EGFR activation for protection against colitis and gastrointestinal helminths

Nicole Maloney Belle[1], *Yingbiao Ji[1], *Karl Herbine[1], *Yun Wei[2,4], *JoonHyung Park[1], Kelly Zullo, Li-Yin Hung[1,2], Sriram Srivatsa[1], Tanner Young[1], Taylor Oniskey[2], Christopher Pastore[1], Wildaliz Nieves[3], Ma Somsouk[3], and De'Broski R. Herbert[1,2]

[1]Department of Pathobiology, University of Pennsylvania School of Veterinary Medicine, Philadelphia, PA 19140, USA
[2]Division of Experimental Medicine, University of California, San Francisco, San Francisco CA 94110 USA
[3]Division of Gastroenterology at ZSFG, University of California, San Francisco, San Francisco CA 94110 USA
[4]Department of Inflammation and Oncology, Amgen Inc., 1120 Veterans Boulevard, South San Francisco, CA 94080.

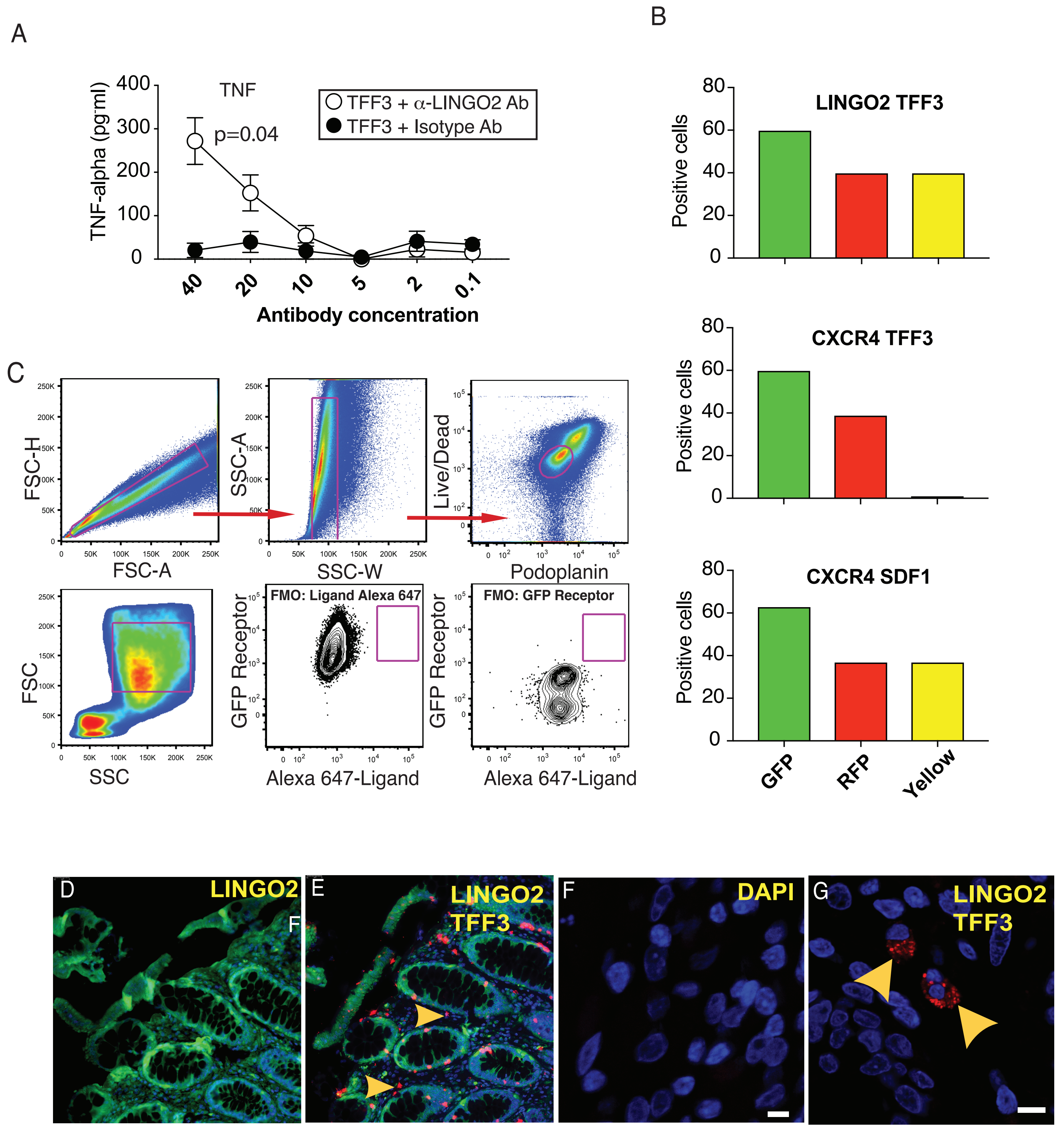

Supplementary Figure 1. LINGO2 interacts with TFF3
(A) TNF alpha release from U937 after rTFF3 treatment (1ug/ml) in the presence of either neutralizing anti-LINGO2 Ab or isotype IgG control at the concentrations indicated. Representative of two independent experiments. (B) Quantification of HEK transfectants that either singly or doubly expressed LINGO2-GFP or CXCR4-GFP transfected (green column) with TFF3-RFP or SDF-1-RFP. This experiment was done by counting the number of cells that were either green, red or yellow, with yellow indicating that the cells transfected had colocalized the two colors, indicative of a possible ligand-receptor interaction. All fluorescent cells on the entire slide were counted. 40x magnification. (C) Gating strategy for the flow cytometry-based ligand binding assay that used HEK cells transfected with receptor-GFP plasmids followed by treatment with Alexa fluor 647 labeled ligands. Data show singlets, live cells and fluorescence minus one (FMO) controls (D) Representative images from proximity ligation assay experiments performed on rectosigmoid biopsy samples from normal human subjects exposed to anti-LINGO2 alone (E) anti-LINGO2mAb and anti-TFF3mAb (20x), (F) DAPI only, and (G) and anti-LINGO2 mAb and anti-TFF3mAb (63x). Scale bar 10 microns. Representative of 3 experiments

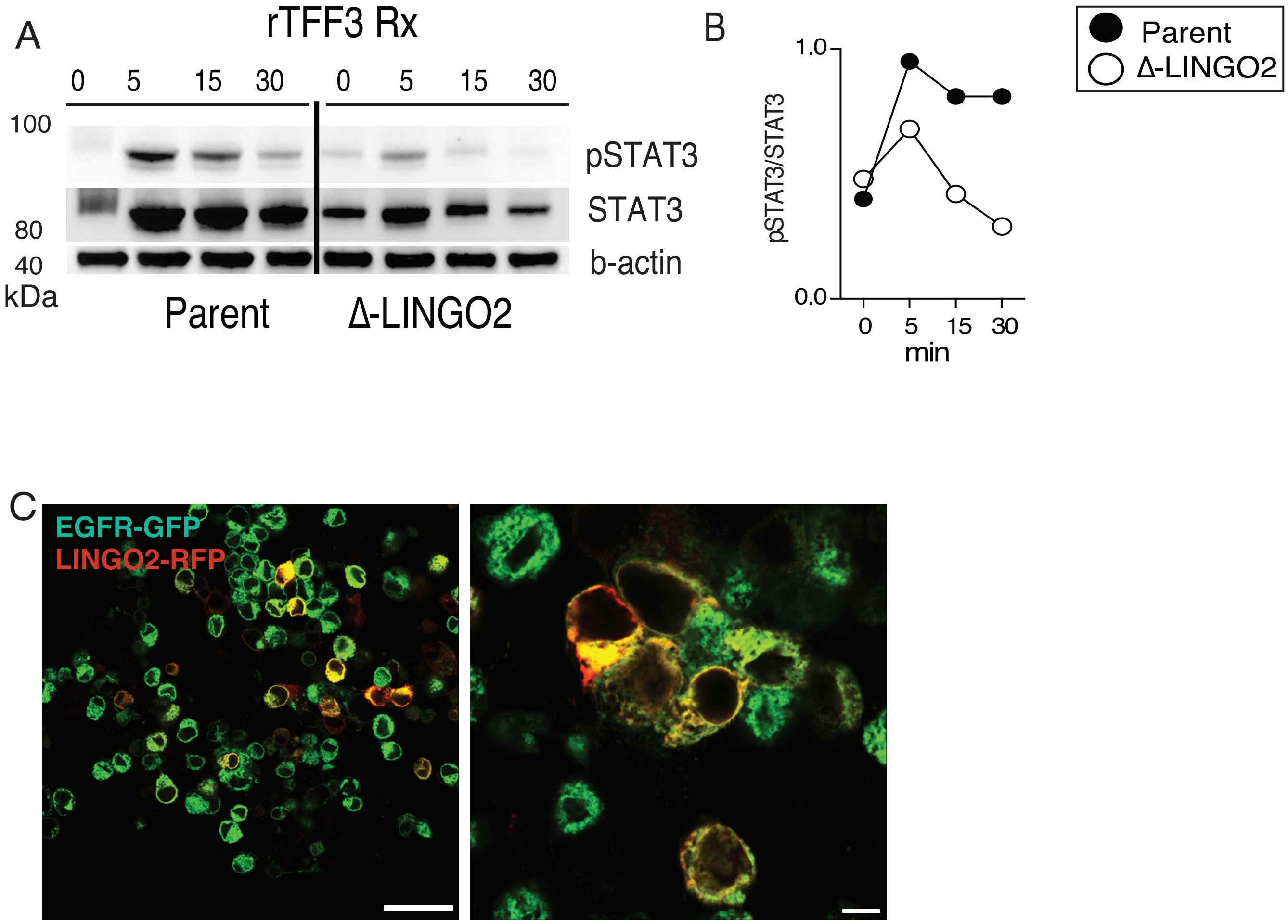

Supplementary Figure 2. LINGO2 binds to EGFR and is required for STAT3 phosphorylation (A)Western blot data shows kinetic analysis of phospo-STAT3, total STAT3 protein and beta-actin loading controls induced by exposure to TFF3-Fc (1 g/mL) within parental MC38 vs.delta-LINGO2 MC38 cells (B) densitometry measurements comparing the ratio of phospo-STAT3 to total STAT3 protein in experiment shown in “A” (C) HEK cells co-transfected with vectors encoding EGFR-GFP and LINGO2-RFP at 4x magnification (left) scale bar 100 microns and 40x magnification (right) scale bar 40 microns

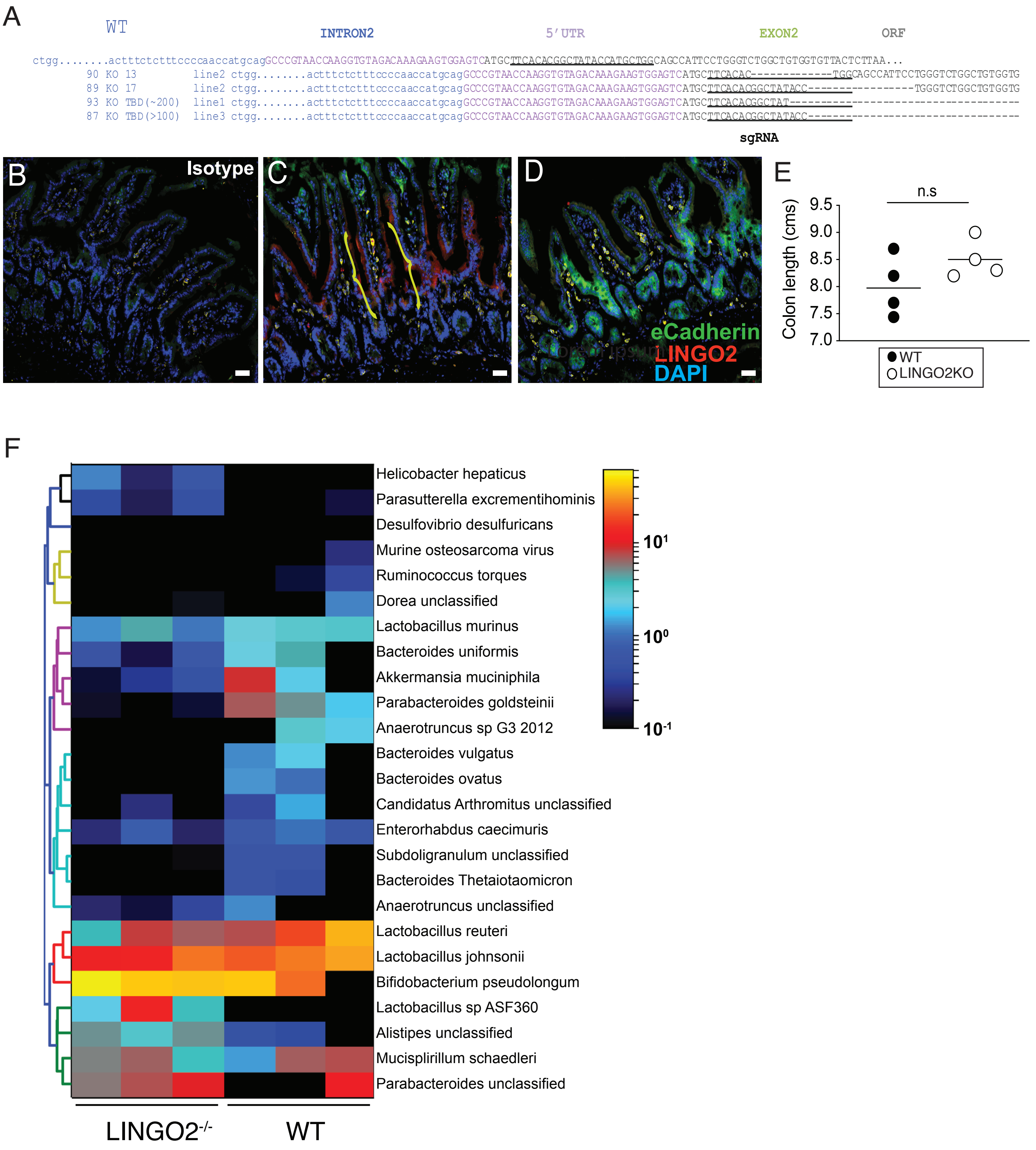

Supplementary Figure 3. Generation of LINGO2KO mice
(A) Genomic sequence of *Lingo2KO* founder lines compared to WT following deletion by CRISPR/CAS9 gene-editing. Representative immunofluorescence images of ileum samples from WT or *Lingo2KO* C57BL/6 mice following co-staining with IgG isotype mAb (B) or anti-LINGO2 Ab and anti-cadherin Ab (20x) (C-D). Scale bar represents 40 microns. Brackets indicate region of LINGO2 signal. Representative images are shown. (E) Colon lengths of naïve WT vs *Lingo2KO* mice. (F) WT and *Lingo2KO* mice have no differences in microbial composition. The heat map is based on 16S rRNA marker gene sequencing of fecal pellets generated using QIIME v. 1.8. OTUs (rows) and individual mice (columns) are clustered by phylogenetic relationship.

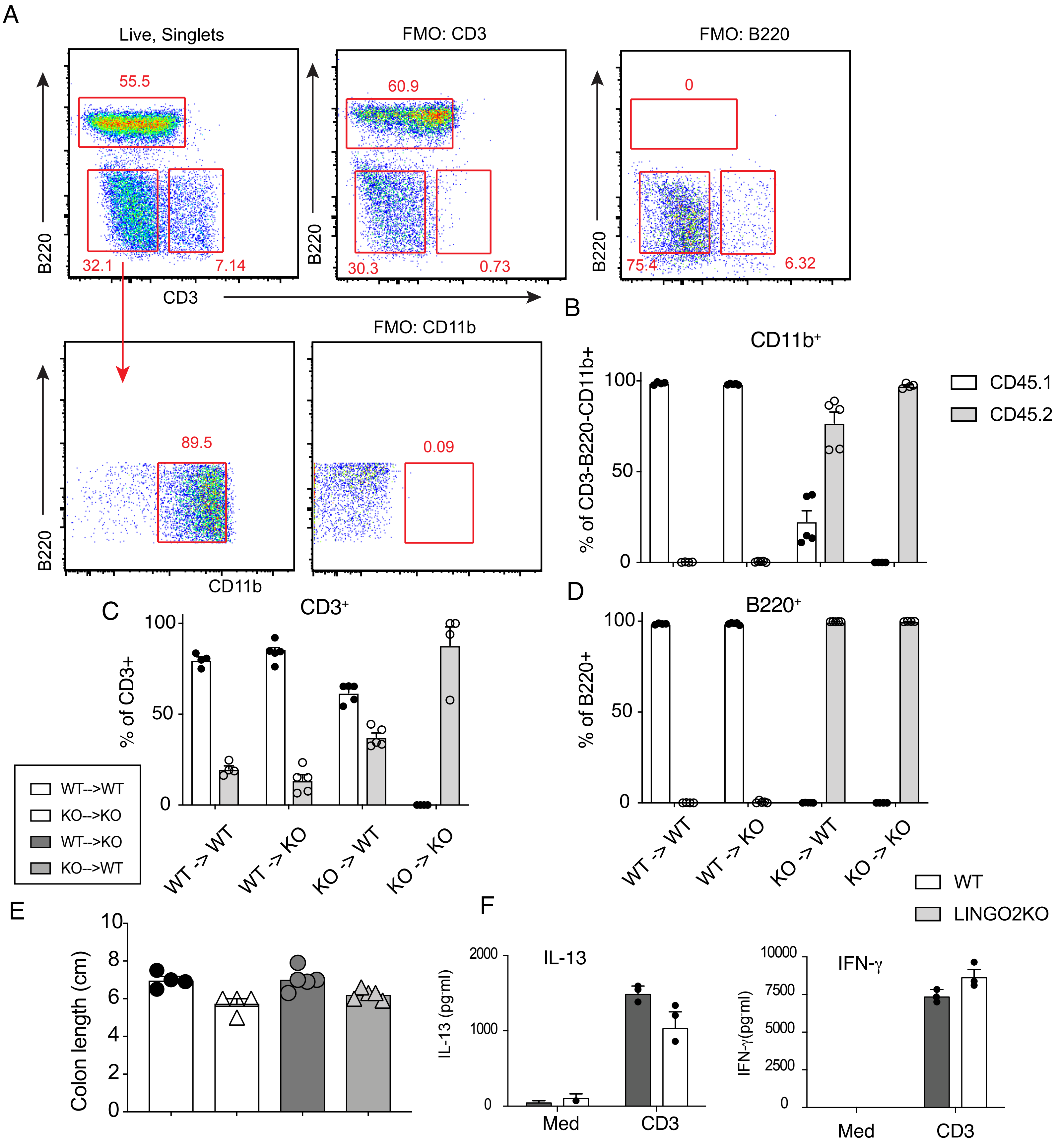

Supplementary Figure 4. Chimera efficiency and cytokine production in WT and LINGO2KO
(A) WT CD45.1 and Lingo2KO CD45.2 mice were irradiated (10Gy) using split dose and reconstituted with 1-2x106 total BM cells and evaluated 6 weeks later for chimerism efficiency showing fluorescence minus one (FMO) controls for CD3, B220,and CD11b. (B-D) Flow cytometry data showing percent donor to recipient ratios for CD11b, CD3 and B220. (E) Day 7 colon lengths following treatment with 2.5% DSS in the drinking water. (F) Cytokine secretion levels for IL-13 and IFN-gamma from naïve mesenteric lymph node cells from WT and Lingo2KO mice following exposure to media only or anti-CD3 mAb (1ug/ml) for 48 hrs.
Data show mean +/- SE of quadruplicate wells from 4 mice/group. Representative of 2 independent experiments.
